# Sub-microWatt threshold nanoisland lasers

**DOI:** 10.1038/ncomms9276

**Published:** 2015-09-22

**Authors:** Hoon Jang, Indra Karnadi, Putu Pramudita, Jung-Hwan Song, Ki Soo Kim, Yong-Hee Lee

**Affiliations:** 1 Department of Physics, Korea Advanced Institute of Science and Technology (KAIST), Daejeon 305-701, Republic of Korea; 2 Convergence and Components & Materials Research Laboratory, Electronics and Telecommunications Research Institute, Daejeon 305-701, Republic of Korea

## Abstract

Ultralow threshold nanolasers have been sought after as power efficient light sources in photonic integrated circuits. Here a single-cell nanobeam laser with a nanoisland quantum well is proposed and demonstrated. Continuous operation at 1.5 μm is achieved at room temperature with an ultralow lasing threshold of 210 nW in absorbed power. The size of the active medium is reduced to 0.7 × 0.25 × 0.02 μm^3^ by removing the absorptive quantum well region surrounding the central cavity. Relatively thick (420 nm) InP slabs are employed to improve the thermal and mechanical characteristics. The nanoisland-based structures will provide a new platform to engineer fundamental light–matter interactions by controlling the size and the location of the nanoemitters, allowing the realization of highly efficient nanophotonic devices.

## Abstract (web summary)

Low threshold power is required for the application of lasers in photonic integrated circuits in order to reduce power consumption. Here Jang *et al*. propose and demonstrate a nanoisland quantum well laser operating at room temperature with an ultralow threshold of 210 nW absorbed at 980 nm.

## Graphical abstract

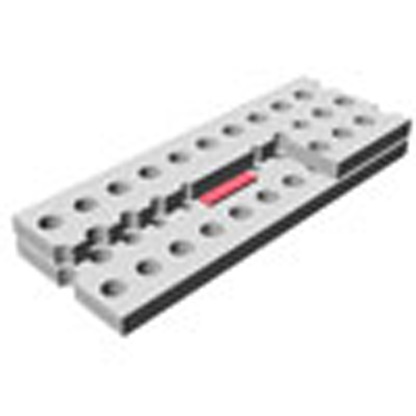

Driven by the explosive growth in data throughput on networks, various optical devices have been developed and deployed^1^,^2^. Optical devices have potential benefits due to their low power consumption through minimal joule heating in the system. Nevertheless, their operating energy has only begun to be competitive with that of pre-existing electrical interconnects, which has recently been demonstrated using free-standing photonic crystal (PhC) lasers^3^. For such application of a laser as a light source in photonic integrated circuits, one needs to lower the threshold to reduce the power consumption that is wasted through heat. Furthermore, minimal heat generation is crucial in a free-standing microlaser to enable continuous wave (CW) operation at room temperature (RT), since efficient heat transfer is generally more difficult without a direct heat sink^4^.

In efforts to fabricate a device that will absorb less energy and generate less heat in the active layer, quantum dots (QDs) have been a popular choice of gain medium^5^,^6^,^7^. The small volume of QDs enables a very low threshold of around 300 nW in absorbed power at RT. However, a QD laser emitting in the telecom wavelength ∼1.5 μm has not yet been reported. A microlaser operating in RT CW at 1.5 μm was first demonstrated by Nozaki *et al*.^8^ in 2007 using small PhC cavities such as H0 and H1 in InGaAsP quantum well (QW) wafers. In that study, they achieved a low effective threshold of 1.2 μW as well as a large spontaneous emission factor of 0.94.

Choice of slab material also plays a crucial role in helping thermal relaxation to succeed in RT CW operation. Martinez *et al*.^9^ in 2009 used an InP-based L7 cavity with a single layer of InAs/InP quantum wires to demonstrate RT CW lasing. In 2011, coupled cavities with 4 InAsP/InP QWs were used by Huang *et al*.^10^ to successfully demonstrate an RT CW PhC laser with enhanced far-field directionality. In both cases, they took advantage of the InP material, which has the high heat conductivity necessary to reduce the thermal resistance of the structures. However, the large sizes of the cavities naturally led to relatively high-effective lasing thresholds of 22 and 14.6 μW, respectively.

Since 2010, one NTT group has reported several sets of results^3^,^11^,^12^ that included RT CW operation with low thresholds using buried heterostructures; one of these studies featured a threshold that was as low as 1.1 μW in absorbed power. Such performance is possible mainly due to the very small active volume surrounded by InP. However, it requires wafer regrowth processes and very precise alignment techniques that are inherently non-trivial.

In the conventional PhC laser based on uniform QW medium, the whole wafer plane stays absorptive until it reaches the transparency condition through pumping. Therefore, in general, one has to pump over an area sufficiently larger than the mode of interest. This results in a non-negligible increase in the lasing threshold. If the absorptive QW background can be properly removed, then a smaller size of active medium should lead to a lower threshold because it will require a smaller number of electron-hole pairs to reach the transparency condition. In practice, however, the total quality (*Q*) factor is commonly determined by fabrication imperfections, which sets the lower bound of the active volume to compensate for the total losses. Therefore, one needs to optimize the size of the QW area to take full advantage of the nanoisland QW structure.

Here we employ a selective wet etching technique^13^ to control the removal of QW layers in the middle of the InP slabs. As a preliminary test, we take a conventional L3 cavity^14^,^15^ in a 420-nm-thick PhC slab of InP that contains a single layer of InGaAsP QW. H_3_PO_4_:H_2_O_2_:H_2_O=2:1:10 solution is used to selectively remove the QW region without touching the top and bottom InP claddings. Depending on how much we etch it away, we can selectively remove the QW outside the cavity region. Figure 1 confirms that there is no QW left outside the cavity when the wet etching is done. One can even see the size of the remaining QW in the images. To confirm the influence of the size of the remaining QW, the threshold of the L3 cavity is studied as a function of the wet-etching time. It is shown in Supplementary Figs 1 and 2 and in Supplementary Note 1 that the threshold power can decrease after the wet-etching processes despite the increase of the optical losses. This preliminary test confirms that selective QW-etching techniques can be used to lower the threshold of PhC lasers by removing the absorptive QW background. In this work, we propose a nanobeam laser based on a self-aligned nanoisland QW active medium. CW lasing is demonstrated at RT with an ultralow threshold of 210 nW that is absorbed at 980 nm.

## Results

### Design and fabrication of nanoisland lasers

**Figure 2:**
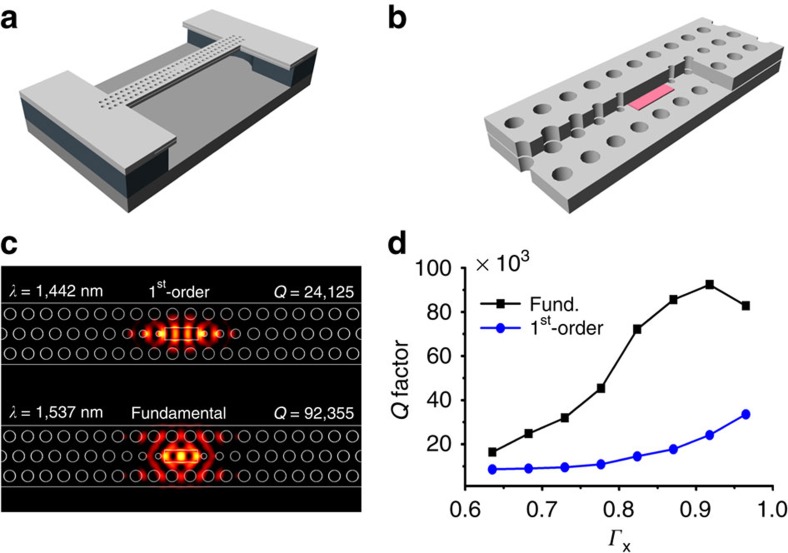
A single-cell nanobeam design for a nanoisland laser. (**a**) Schematic of the nanobeam design for a single-cell cavity. (**b**) A magnified view near the cavity. A part of the upper InP cladding is cut to show the small active region (coloured) confined inside the cavity. (**c**) |**E**|^2^ field profile of the fundamental and the first order modes in the current design. (**d**) *Q* factor versus QW confinement factor in Γ–K direction in the single-cell cavity.

First, we adopt cavity structures that mainly feature a size of one to three lattice constants (L1 to L3) along the Γ–K direction in a three-stripe triangular PhC lattice with periodicity around 390 nm. While the device is aimed to keep the advantages of a 1D (one-dimensional) structure as much as possible^4^,^16^,^17^, we choose the three-stripe structure to control the wet-etching speed for QW isotropic in every direction so that the remaining QW area resembles the shape of the cavity design. The proposed structure for a single-cell cavity (L1), for example, is shown in Fig. 2a; the magnified view near the cavity is shown in Fig. 2b. In our design, we slightly modulate the lattice constant and the air-hole radius near the cavity to suppress the Fourier components of the cavity mode inside the radiation zone. The single-cell cavity has an advantage to achieve a single-mode operation due to its wider-free spectral range so that only one mode falls into the gain bandwidth. The choice of the small cavity, however, reduces the modal gain, especially after isolating the small active medium inside the cavity; one might now wonder how large *Q* factor would be required for such device to meet the lasing condition. In fact, the minimum *Q* factor that is required to reach the threshold is inversely proportional to the confinement factor of the QW inside the cavity, rather than the QW size itself. We show in Supplementary Note 2 that the minimal *Q* factor for lasing in the present design is estimated to be around 3,000.

Finite-difference time-domain calculations confirm that the *Q* factor of the fundamental mode of the single-cell cavity can be over 92,000, which is much larger than the minimal *Q* factor required for lasing. The fundamental mode is spectrally 100 nm apart from the first-order mode in the simulation. The corresponding |**E**|^2^ field profiles are presented in Fig. 2c. The larger cavities such as L2 and L3 are readily able to achieve larger *Q* factors over 300,000 and 700,000, respectively, in the simulations. It should be noted, however, that the location of the boundary of the remaining QW area (that is, index discontinuity) relative to the anti-nodes of the resonant modes sensitively influences the cavity losses. The *Q* factors of the fundamental and first-order modes are shown in Fig. 2d as functions of the confinement factor (*Γ*_*x*_) of the QW along the oscillation (that is, Γ–K) direction. The peak near *Γ*_*x*_=0.92 can be understood as the Fabry–Perot effect from the sidewalls of the remaining QW, which resonantly enhances the field inside when the anti-node lies at the boundary. Fine control of the QW size inside the cavity can be achieved by tuning the size of the holes near the cavity and the wet-etching time.

The designed nanobeam structures are built in a 420-nm-thick InP slab that contains a 7-nm-thick InGaAsP single QW sandwiched by two 6-nm-thick InGaAsP barriers. This choice of single QW not only minimizes the active volume, but also maintains the high *Q* factor after QW etching^18^. One notable aspect of our structure is that we employ a slab that is thicker than the lattice constant. Thick slabs have advantages in improving mechanical stability^19^ and thermal dissipation. In reality, however, it is rather unusual to use thick slabs^20^,^21^ because the propagating modes of the transverse magnetic (TM)-like band come down spectrally close to the transverse electric (TE)-like resonant mode. In our nanobeam design, the 1D-like characteristic largely reduces the number of allowed propagating modes in the structure such that the number of channels coupling to the TM-like modes can be minimized (Supplementary Fig. 3). This can enhance tolerance to fabrication errors (that is, asymmetries) such as the non-vertical etching slope in the thick slabs.

**Figure 3:**
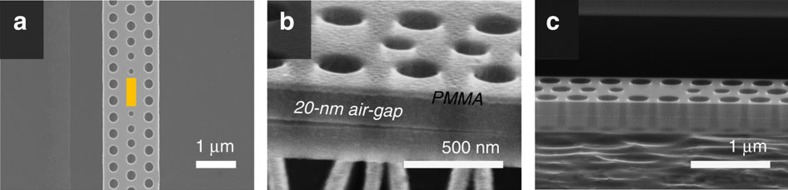
SEM images of a nanoisland laser. (**a**) The colored area describes the size of remaining QW after the wet etching. (**b**) An SEM image after the wet etching, where the thin layer on top of the structure corresponds to the PMMA layer to protect the upper InP cladding during the wet-etching process. The wet etching leaves a 20 nm air-gap between the InP claddings. (**c**) An SEM image of the final structure.

The fabrication procedure is similar to the series of standard nanofabrication processes described in the previous reports^13^,^22^. First, the nanobeam designs are patterned using electron-beam lithography with PMMA on top; designs are then transferred by Cl_2_ assisted Ar ion-beam etching onto the InP slab that contains a single layer of InGaAsP QW at its center. This is followed by the wet-etching process, in which an InGaAs sacrificial layer is removed using the H_3_PO_4_:H_2_O_2_:H_2_O acid solution. At the same time, the InGaAsP QW layer is also partially etched away, leaving the gain only inside the cavity region, as described in Fig. 3a.

Figure 3b shows that we still keep the polymer (PMMA) mask on the structure during the wet-etching process as a protection layer to protect the top InP cladding, since the selectivity of the etchant over InP/InGaAs(P) is, in fact, not infinite. In our case with a relatively thick PhC slab, it is important to preserve vertical symmetry of the structure because the *Q* factor of a TE-like cavity in a thick slab is generally vulnerable to such asymmetry as the TM-like band comes down closer to the TE-like cavity mode. Furthermore, one can also notice in Fig. 3b that the thin air-gap between the two InP claddings often collapses outside the cavity. Although this collapsing may slightly reduce the radiation loss due to a red-shift (∼24 nm) of the cavity resonance (Supplementary Fig. 4), the 20 nm air-gap is so thin that either its existence or its collapsing has little influence on the performance of the device (Supplementary Figs 5 and 6, Supplementary Note 3). Last, the PMMA mask is removed using O_2_ plasma ashing. This series of processes results in an InP-based PhC structure on a 420-nm-thick air-bridge with QW confined in the cavity region. A scanning electron microscope (SEM) image of the final nanobeam structure is shown in Fig. 3c.

### Measurement of lasing characteristics

**Figure 4:**
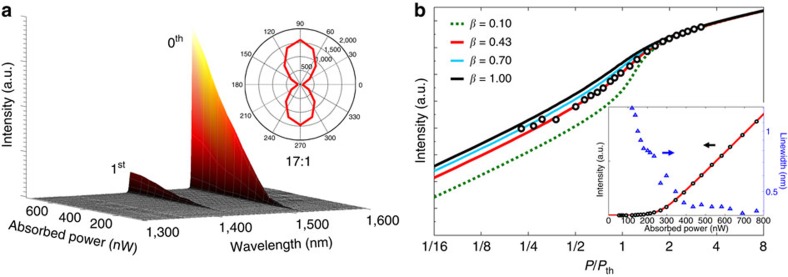
Optical characterization of a nanoisland laser. (**a**) Photoluminescence spectrum of the nanobeam as a function of absorbed power. The inset describes the polarization of the lasing mode. (**b**) Output power of the lasing mode is plotted in logarithmic scale. The inset shows the same data in linear scale with linewidth as a function of absorbed power, which proves the existence of the threshold around 210 nW.

Laser emission from the fabricated sample was characterized by pumping the sample with a 980 nm laser diode at RT in CW condition. A microscope objective lens (× 50) with numerical aperture (NA) of 0.85 was used to focus the pump beam onto a spot as small as 1.2 μm in diameter (Supplementary Fig. 7) and to collect the emitted light from the sample at the same time. Figure 4a shows the laser spectra of a sample with the single-cell cavity as a function of absorbed power. In the spectrum, only two cavity modes appear over a wide range of 300 nm, while only one peak that corresponds to the fundamental mode efficiently lases near 1,520 nm at the incident power of ∼7.8 μW.

Here the pump power incident on the remaining QW area is estimated to be around 27% of the total power carried by the Gaussian beam. With an absorption coefficient *α*=14,000 cm^−1^ at 980 nm, the absorbed power in the QW at the threshold is estimated to be 210 nW, which is roughly 10% of the incident power on the active area around the threshold. In fact, the absorbed power around the threshold turns out to be of the same order of magnitude as the power required to satisfy the transparency condition in our structure. This implies that our structure is designed to have a sufficiently high *Q* factor (>3,000, Supplementary Note 2) despite the fact that the selective QW etching leaves the 20 nm-thick air-gap, which may cause additional optical losses.

The inset in Fig. 4a shows well-defined in-plane polarization of the fundamental mode that is perpendicular to the longitudinal (that is, Γ–K) direction of the cavity. This indicates the TE-like characteristic of the resonant mode. The experimental data of the *L*_in_-*L*_out_ curve from the fundamental mode in Fig. 4a is fitted to the rate equations, as shown in Fig. 4b. The best fitting is obtained when the spontaneous emission factor (β) is bigger than 0.4, with a gain coefficient *g*_0_∼3,000 cm^−1^ and a *Q* factor of 12,000. The parameters and their values can be found in Supplementary Table 1, while the rate equations are shown in Supplementary Note 4. The inset presents the same data but in linear scale, with a change of measured linewidth that clearly shows the transition from spontaneous emission to stimulated emission. The resolution bandwidth of our monochromator (CVI DK480) is limited to ∼0.4 nm, which only provides a lower bound of the *Q* factor in this case.

In our additional cavity designs such as L2 and L3, there exist several resonant modes inside the gain bandwidth due to the longer cavity sizes. Despite the competition between the different modes, lasing is observed in both cavities L2 and L3 with very low thresholds of 520 and 930 nW, respectively. The measurement data for L2 and L3 are presented in Supplementary Fig. 8, while we focus on the single-cell cavity here.

The CW operation remains stable at least three times above the threshold at RT, which essentially relies on the thermal stability of the device. In the current structure, heat generation is reduced due to the very small active area confined inside the cavity. Furthermore, the use of thick InP material with high thermal conductivity of 68 Wm^−1^ K^−1^ effectively reduces the thermal resistance of the device^3^,^11^.

To confirm the thermal stability of our device, we estimated the temperature change in the active region by measuring the shift of the lasing wavelength when we gradually changed from a pulsed regime with 50 ns (1% duty) to CW operation. In all cases, the peak power of the pump was fixed at around 20 μW, which is above the thresholds. As shown in Supplementary Fig. 9, the lasing wavelength in CW operation is observed to be red-shifted around 0.5 nm with respect to the pulsed regime. The dependence of the lasing wavelength on the temperature in the InGaAsP/InP structure was also measured and found to be around 0.11 nm K^−1^ (ref. 11). Therefore, we estimate that the temperature increase in the active region in CW operation is around 5 K for an input power of 20 μW.

## Discussion

In summary, we have proposed and demonstrated a nanoisland QW nanobeam laser in RT CW operation. Such RT CW operation is possible due to the ultralow threshold power of 210 nW absorbed at 980 nm, as well as to the relatively high-heat conductivity of the InP material. The unique structure based on the nanoisland QW facilitates control of the size and the location of the nanoemitters, thereby showing high potential for use in the fundamental studies on light–matter interaction in microscopic systems, and in the realization of power efficient nanophotonic devices.

When employed for electrical pumping^3^,^17^,^23^,^24^, our nanoisland QW structure may offer an ideal platform to realize sub-microampere threshold nanolasers at RT because this structure has a thin air-gap that can be used as electrical insulation between the p-doped and n-doped claddings. If one manages to prevent the air-gap from collapsing, then it can fundamentally limit the leakage current, which would otherwise flow outside the cavity region. Besides, our structure does not require a current post at the center^17^,^23^, of which existence can degrade the quality factors of the cavity modes and limit the choice of alternative cavity designs.

## Methods

### Wafer structure

Nanoisland lasers were fabricated using a (420-nm thick InP slab)/(1.5-μm thick InGaAs sacrificial layer)/(*n*-InP substrate) wafer structure. A single layer (7 nm) of InGaAsP QW sandwiched by two barriers (6 nm, each) was embedded in the middle of the InP slab.

### Wet etching

H_3_PO_4_:H_2_O_2_:H_2_O=2:1:10 solution was used to selectively remove the InGaAsP QW region without touching the top and bottom InP claddings. To increase the selectivity, we performed the wet etching at 0–4 °C.

### Photoluminescence measurements

The fabricated nanoisland laser was characterized by pumping with a 980-nm laser diode at RT in CW condition with no intentional cooling. We used a microscope objective lens (× 50) with NA of 0.85 to focus the pump beam and to collect the emitted light from the sample at the same time. The spot size at the beam waist was as small as 1.2 μm in diameter; measurement results using the knife-edge method are presented in Supplementary Fig. 7.

## Additional information

**How to cite this article:** Jang, H. *et al*. Sub-microWatt threshold nanoisland lasers. *Nat. Commun.* 6:8276 doi: 10.1038/ncomms9276 (2015).

## Supplementary Material

Supplementary Information Supplementary Figure 1-9, Supplementary Table 1, Supplementary Notes 1-4 and Supplementary References.

## Figures and Tables

**Figure 1:**
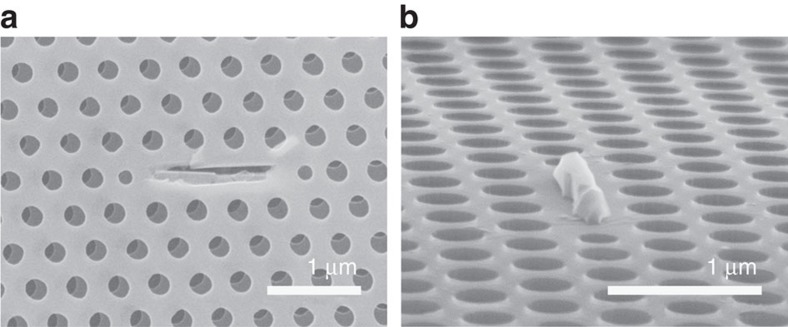
No QW left outside the cavity after the selective wet etching. (**a**) Carefully breaking the sample after the wet etching, we observe that dislocation between the upper and lower InP cladding appears only outside the cavity, which proves that QW remains only inside the cavity region. (**b**) Mechanically removing the upper InP cladding, a broken piece of InP remains attached to the corresponding QW area that is left after the wet etching.
