# Supplementary material for: Sub-microWatt threshold nanoisland lasers
Source: Nat Commun. 2015 Sep 22;6:8276. doi: 10.1038/ncomms9276 (PMC4595645; doi:10.1038/ncomms9276)
Supplement: Supplementary Information — Supplementary Figure 1-9, Supplementary Table 1, Supplementary Notes 1-4 and Supplementary References. [file ncomms9276-s1.pdf]

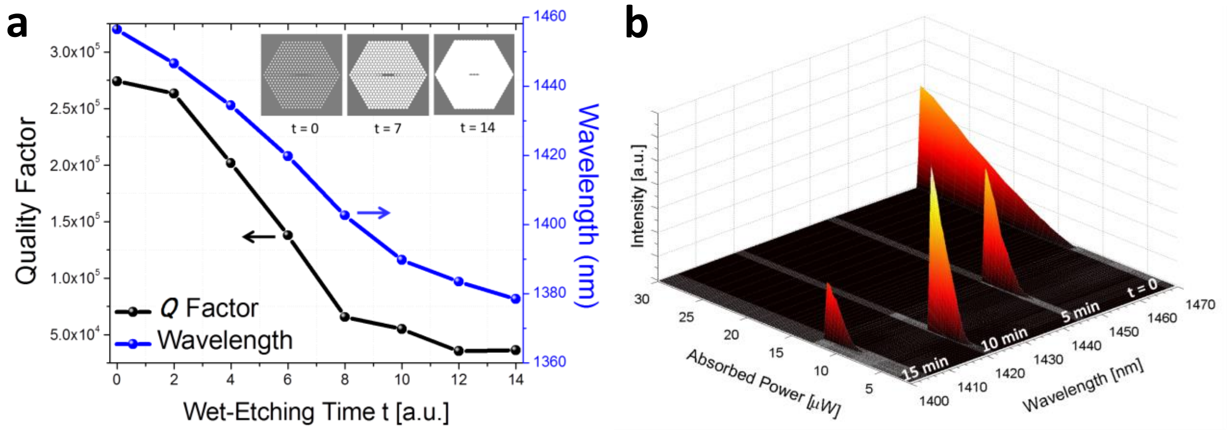

Supplementary Figure 1 | Thresholds of the L3 cavity as a function of wet-etching time

(a) Change of a *Q* factor (black line) and a resonant wavelength (blue line) of a typical L3 cavity are calculated as a function of wet-etching time. The inset describes the removal of QW during the wet-etching in the simulation. The grey color corresponds to the remaining QW area. **(b)** Four sets of laser spectra are plotted as a function of the absorbed power. Each set corresponds to the different wet-etching time, i.e. 0 min, 5 min, 10 min and 15 min from the right-hand side. The pump laser in this preliminary test was in a pulsed regime at 980 nm with 5% duty cycle and the repetition rate of 1MHz.

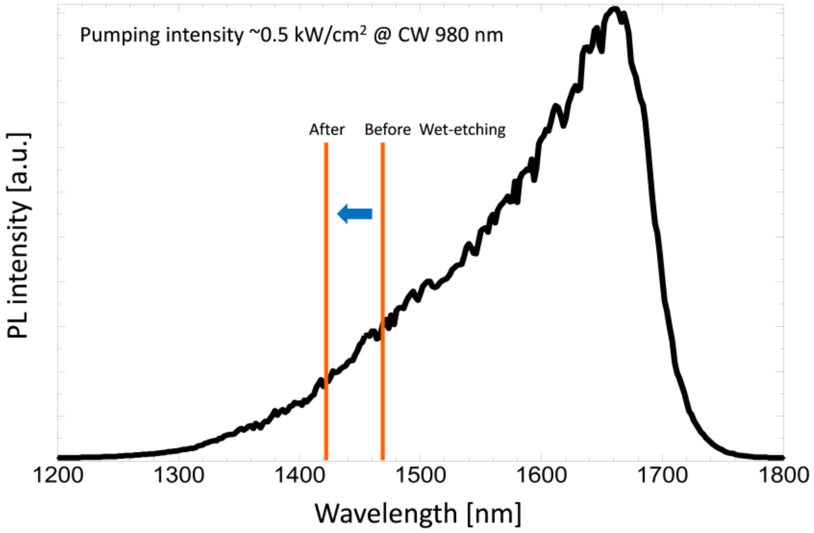

Supplementary Figure 2 | Photoluminescence (PL) spectrum of our InGaAsP QW

PL spectrum of our QW wafer was measured to predict the change of the optical gain in shorter wavelength. In this case, the incident pump power was ~ 50 μW at 980 nm in CW condition with a beam radius of ~ 2 μm. The two colored lines describe the blue-shift of the cavity mode, which was shown in Supplementary Figure 1.

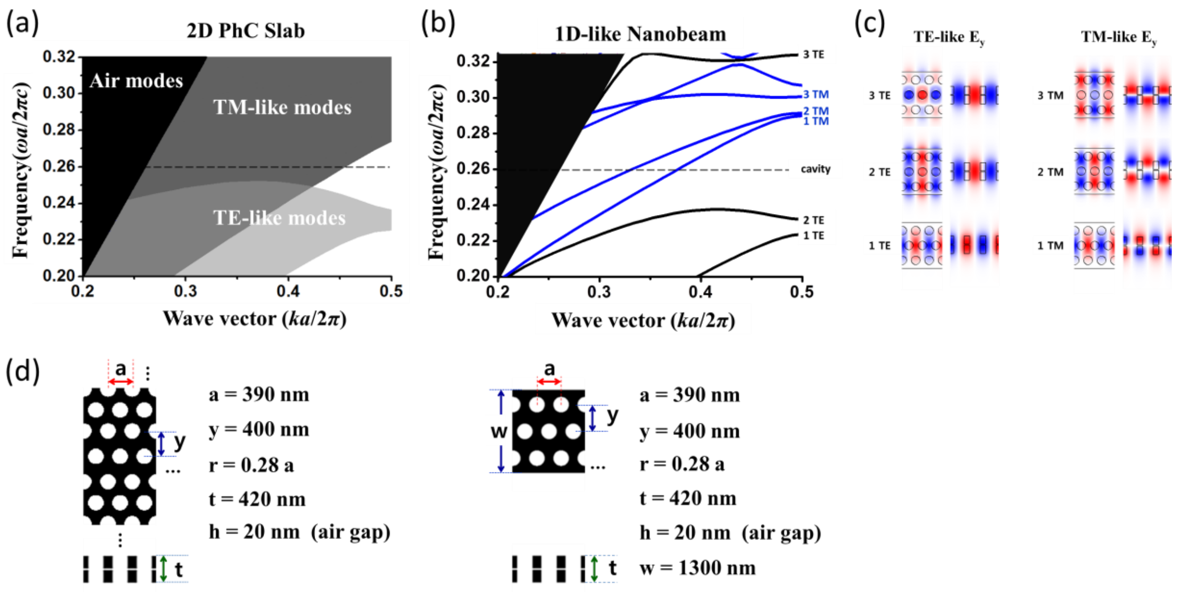

Supplementary Figure 3 | Photonic band structures in 2D PhC slab and 1D-like nanobeam

This is a comparison between photonic band structures in **(a)** 2D PhC slab and **(b)** 1D-like Nanobeam in a 420 nm InP slab with 20 nm air-gap in the middle. The black curves correspond to TE-like modes, and the blue curves TM-like modes. The dashed line represents the location of the cavity mode around 1500 nm. Here, we focus on the modes that have the same horizontal parities as the TE-like cavity modes, since the possible fabrication errors in the thick slabs are mostly the vertical asymmetries such as non-vertical etching slope. The E_y field profiles of the corresponding modes are shown in **(c)**. These TM-like modes are the potential leakage channels when the structure bears the vertical asymmetries such as non-vertical etching slope in the thick slabs. However, the number of these allowed propagating states is largely reduced in the 1D-like nanobeam structure compared with the 2D PhC structures, as shown in **(a)** and **(b)**. This improves the tolerance to the fabrication imperfection. The structural parameters used in the simulations in **(a)** and **(b)** are presented in **(d)**. Note that we slightly increased the line space (y) around the central strip in our designs. This is later to help increase the QW confinement factor inside the cavity after wet-etching.

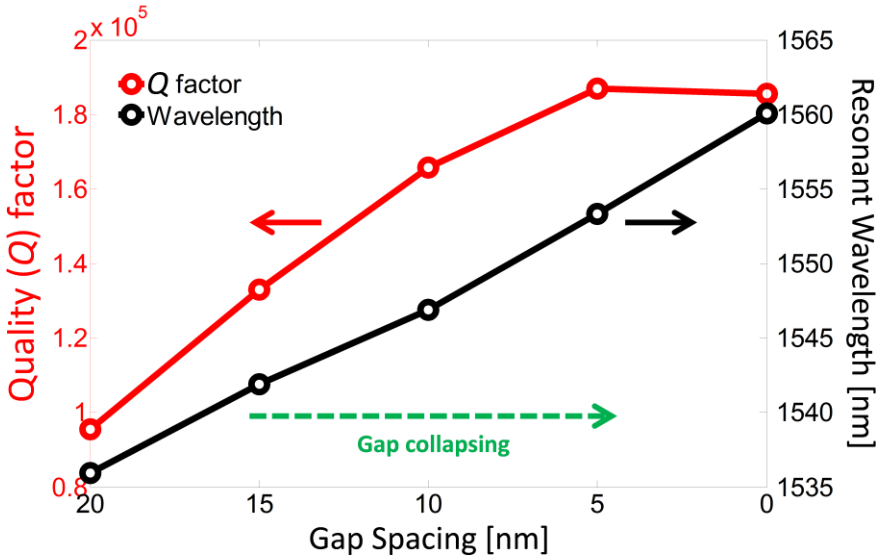

Supplementary Figure 4 | Change of the *Q* Factor and the lasing wavelength as a function of gap spacing

As the air gap between two InP claddings collapses (and the gap spacing accordingly decreases), the resonant mode shifts to a lower frequency (black line), moving away from the light cone. This consequently increases the *Q* factor (red line) of the structure. In the simulation, we fix the thickness of both upper and lower InP claddings (200 nm, respectively).

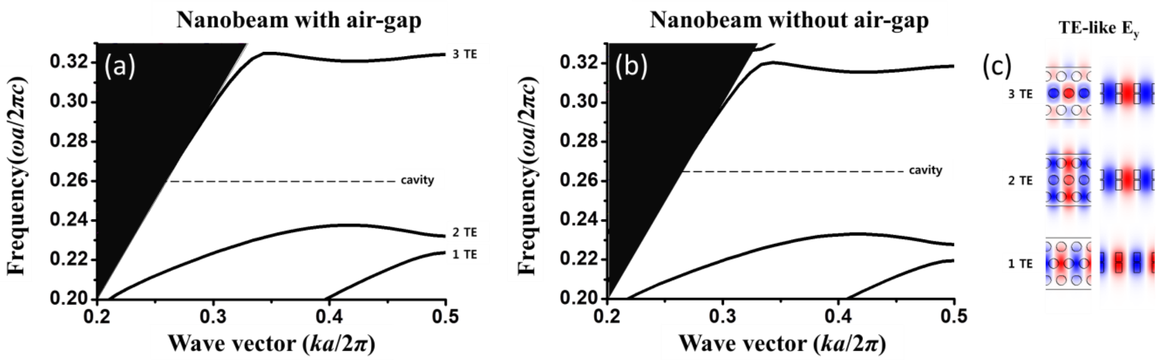

Supplementary Figure 5 | Photonic band structures with and without the air-gap

Photonic band structures of the 3-stripe nanobeam structure with **(a)** an air-gap and **(b)** no air-gap are calculated. Here, we fix the thickness of the two InP claddings (200 nm, respectively). The dashed lines in both pictures represent the location of the cavity mode around 1500 nm. The small blue-shift of the cavity mode after gap-collapsing is taken into account in **(b)**. The E_y field profiles of the corresponding modes are shown in **(c)**.

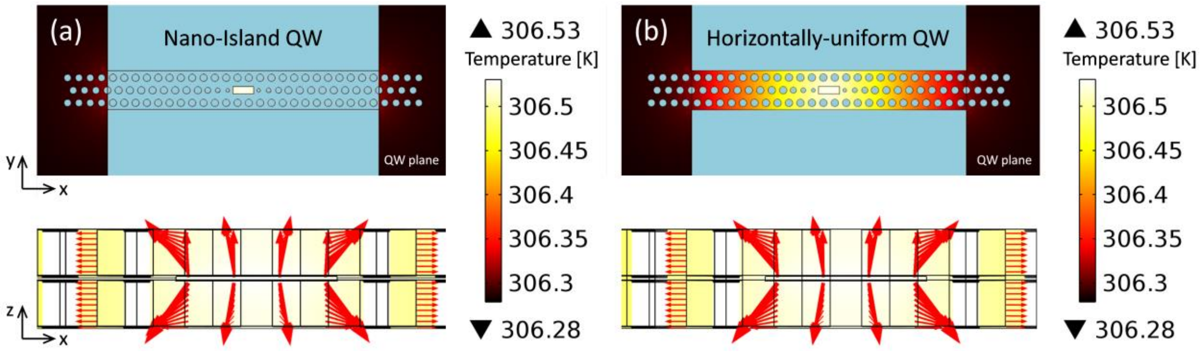

Supplementary Figure 6 | Thermal dissipation in the structure with or without the air-gap

The temperature of **(a)** the nano-island laser with an air-gap and **(b)** the conventional nanobeam laser with no air-gap is calculated. In the COMSOL 3D calculations, a 2 μW heat source is located in a 0.7×0.25×0.02 μm^3 active medium, and no heat sink under the substrate is assumed. The arrows (red) represent the direction and the magnitude of thermal flux in the device. The temperature increase in the active medium is calculated to be 13.38 C°, which is practically the same for both **(a)** and **(b)**. The temperature difference between the two cases appears when the generated heat is large over 20 μW.

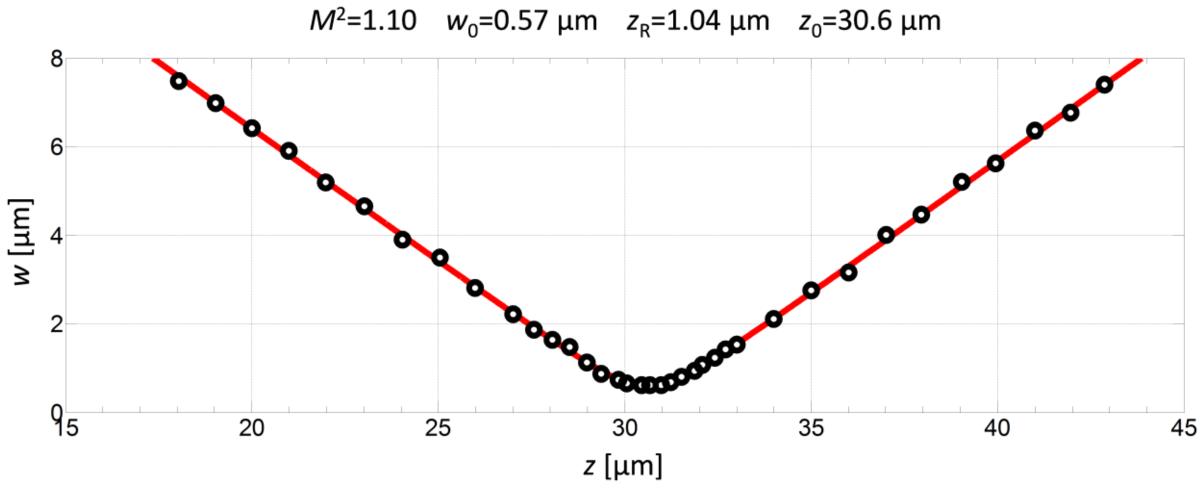

Supplementary Figure 7 | Measurement of the beam radius using the knife-edge method

The beam radius was measured along the beam propagation direction. The radius at the beam waist (w_0) is estimated to be around 0.6 µm. The corresponding Rayleigh length $(2z_R)$ is 2.1 µm. In the measurements, the pump laser beam at 980 nm was focused using objective lens (× 50) with NA of 0.85. We used a sharp edge of a cleaved silicon wafer that was coated with gold on top to partially block the pump beam, and measured the transmission power while moving the edge along the perpendicular direction to the beam propagation to scan the beam profile. We conveniently take the points of 16% and 84% transmission, which are separated by a distance of the beam radius w. This method would give an exact result only for a beam with $M^2 = 1$, whereas the error is not large when M^2 is low. And then, we repeated the same measurement for each beam cross-section along the beam propagation to determine the focus and the divergence of the beam. The data is fitted to the equation $w(z) = w_0\sqrt{1+\left(\frac{M^2\lambda}{\pi w_0^2}z\right)^2}$ and plotted above.

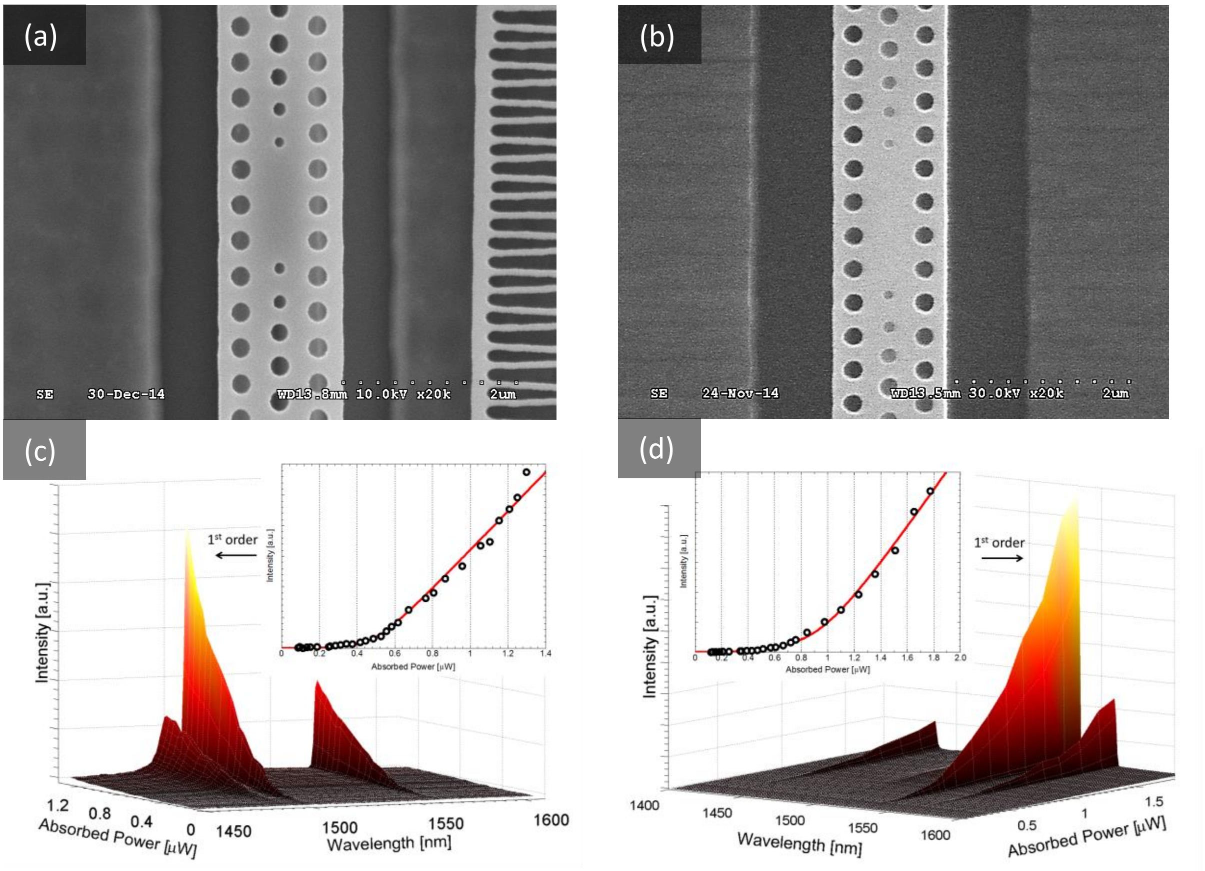

Supplementary Figure 8 | The measurement data for the 3-stripe L2 and L3 cavities

In addition to the single cell cavity described in the main text, we investigated other cavity designs including the ones that feature double (L2) and triple (L3) cells along the Γ-K direction in the 3-stripe triangular PhC lattice with periodicity around 390 nm. The SEM images of the final structure of **(a)** L2 and **(b)** L3 cavity are presented here. The bigger cavities provide larger modal gain together with larger Q factors over 300,000 and 700,000 in the simulations for L2 and L3 respectively, whereas, there exist several resonant modes inside the gain spectrum due to the longer cavity sizes. Photoluminescence spectra of the nanobeams as a function of absorbed power are plotted for **(c)** L2 and **(d)** L3 cavity. The insets show the linear plot of ηL_{in}- L_{out} curve of the strongest mode in each cavity. Despite the competitions between the different modes, lasing was observed with very low thresholds of 520 nW and 930 nW, respectively. Interestingly, however, sometimes it is the 1st-order mode that turns out to be dominant over the fundamental mode, even though the Q factor (>20,000) of the 1st order mode is lower than that of the fundamental. This is understood as a larger modal gain of the 1st-order mode due to its better overlap with the remaining QW area. The spontaneous emission factor β for the 1st order mode in each cavity is estimated to be $\beta \approx 0.34$ for L2 and $\beta \approx 0.19$ for L3 cavity.

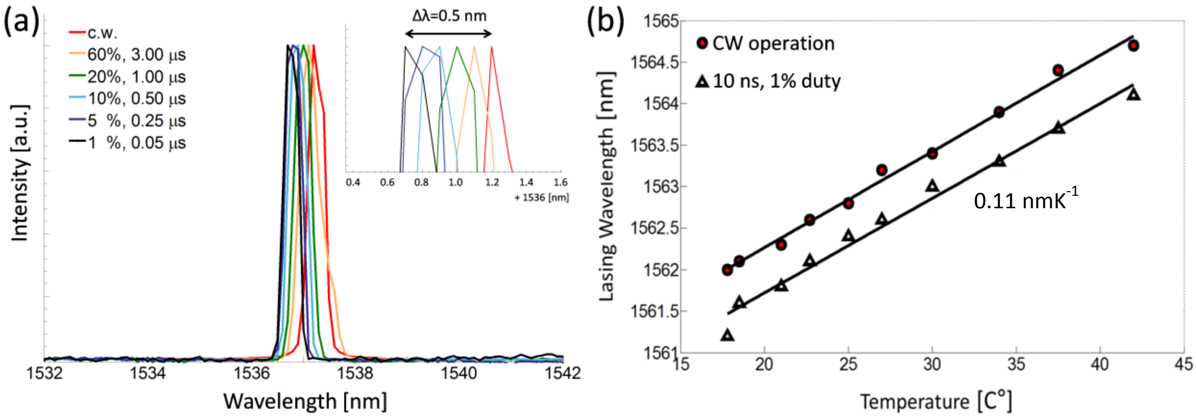

Supplementary Figure 9 | Thermal characteristics of a single-cell nanobeam laser with nano-island QW

We first observed **(a)** the lasing wavelength in a pulsed regime with 1% (black), 5 % (navy), 10% (light blue), 20% (green), 60% duty cycle (yellow), and CW operation (red). To measure the lasing wavelength as a function of duty cycles, the peak power in the pulsed regime was fixed around 20 µW in all cases, which was the incident power in the CW condition. Changing the temperature gradually, we also observed the shift of lasing wavelength as shown in **(b)**. Here, the dependence of resonant wavelength on temperature is estimated to be 0.11 nmK^{-1}. This coefficient is an intrinsic property of material that majorly relies on the change of the refractive index as a function of temperature rather than the structure of the device. This value (i.e. 0.11 nmK^{-1}) is similar to that (0.095 nmK^{-1}) of the LEAP laser (see Supplementary Reference [1]) where they also employed an InP/InGaAsP QW structure. Therefore, we conclude that the shift of the lasing wavelength shown in **(a)** corresponds to the temperature increase of 0.5 nm$\times$0.11 nmK^{-1} = 5.5 K under the incident power of 20 µW.

Supplementary Table

Parameters	Values
Active surface area (A_a)	$1.75 \times 10^{-9}cm^2$
Active volume (V_a)	$3.50 \times 10^{-15}cm^3$
Surface recombination velocity (ν_s)	$3000\ cms^{-1}$
Bimolecular recombination coefficient (B)	$1.0 \times 10^{-10}cm^3s^{-1}$
Auger non-radiative recombination rate (C)	$5.0 \times 10^{-29}cm^6s^{-1}$
Confinement factor (Γ)	8.1×10^{-3}
Gain coefficient (g_0)	$3000\ cm^{-1}$
Transparency current density (N_{tr})	$1.0 \times 10^{18}cm^{-3}$
Q factor (Q)	12,000
Effective refractive index (n_{eff})	2.58
Lasing wavelength (λ_l)	1520 nm
Pump laser wavelength (λ_p)	980 nm

Supplementary Table 1 | The parameters used in the rate-equations in Supplementary Note 4

Supplementary Notes

Supplementary Note 1: Removal of the absorptive QW background in the conventional L3 cavity, and the change of its thresholds as a function of wet-etching time

In the conventional PhC laser based on uniform QW medium, the entire wafer plane stays absorptive until it reaches the transparency condition through pumping. This results in the non-negligible increase in lasing threshold. If the absorptive QW background can be removed properly, a smaller size of active medium should lead to a lower threshold since it requires a smaller number of electron-hole pairs to reach the transparency condition. In practice, however, the total quality (Q) factor is limited, which sets the lower bound of active volume to compensate for the total losses. Therefore, one needs to optimize the size of the QW area to take full advantage of the nano-island QW structure.

To confirm the influence of the size of remaining QW, the thresholds of the L3 cavity is studied as a function of wet-etching time. The calculations in Supplementary Figure 1 (a) shows that the *Q* factor decreases as the QW outside the cavity begins to be etched away leaving a 20 nm thin air-gap that causes an optical loss. However, this now requires a smaller number of electron-hole pairs to reach the transparency condition after removing the absorptive QW background. Indeed, Supplementary Figure 1 (b) shows that the threshold first keeps decreasing down to 37 % of the initial value as we increase the wet-etching time for the QW. And then the threshold eventually increases later when the reduced gain finally cannot overcome the cavity loss. The blue-shift of the lasing wavelength observed in Supplementary Figure 1 (b) agrees well with the calculation shown in (a). Please note that this reduction of the lasing threshold after wet etching is not originated from a larger optical gain in shorter wavelength. The measured photoluminescence spectrum of our InGaAsP QW has its maximum around 1662 nm, as shown in Supplementary Figure 2. Therefore, one can expect that the gain does not increase when the wet-etching makes the cavity resonance blue-shifted near 1500 nm.

This preliminary test confirms that a smaller size of active medium can lead to a lower threshold, provided that optical loss does not significantly increase.

Supplementary Note 2: The minimal *Q* factor for lasing with the single cell cavity

The minimum Q factor that is required for the device to meet the lasing condition is inversely proportional to the confinement factor of the QW. According to the calculations below, the minimal Q factor for lasing with the single cell cavity is estimated to be around 3,000 when the gain coefficient g_0 is assumed to be $3{,}000$ cm^{-1}.

Gain in one-round trip in the cavity:

$$2 \cdot g_{\mathrm{M}} \cdot L_x^{\mathrm{QW}} = 2 \cdot g_0 \cdot \Gamma_y (2\Gamma_z) L_x^{\mathrm{QW}} \tag{1}$$

Loss in one-round trip in the cavity:

$$Loss = \frac{2kL_x^{\mathrm{cav}}}{Q} = \frac{4\pi\ (n/\lambda) L_x^{\mathrm{cav}}}{Q} \tag{2}$$

From the lasing condition Gain = Loss, Q factor required for lasing:

$$\mathrm{Q} = \left(\frac{\pi n}{g_0 \lambda \Gamma_y \Gamma_z}\right)\left(\frac{L_x^{\mathrm{cav}}}{L_x^{\mathrm{QW}}}\right) = \frac{\pi n}{g_0 \lambda \Gamma_x \Gamma_y \Gamma_z} = \frac{\pi n}{g_0 \lambda \Gamma_{\mathrm{QW}}} = \left(\frac{\pi \cdot 3.2}{0.3\ \mu\mathrm{m}^{-1} \cdot 1.5\mu\mathrm{m} \cdot \left(\frac{700}{900}\right) \cdot \left(\frac{250}{600}\right) \cdot \left(\frac{10}{400}\right) \cdot}\right) \approx 2{,}800 \tag{3}$$

The effective cavity size along the direction of oscillation: L_x^{cav}

The size the remaining QW along the direction of oscillation: L_x^{QW}

The material gain coefficient: $g_0 \approx 0.3\ \mu\mathrm{m}^{-1}$

The QW confinement factors: $\Gamma_{\mathrm{QW}} = \Gamma_x \Gamma_y \Gamma_z \approx \left(\frac{700}{900}\right) \cdot \left(\frac{250}{600}\right) \cdot \left(\frac{10}{400}\right)$

Supplementary Note 3: Influence of the air-gap collapsing on the device: comparison between the two cases with and without the air-gap.

As shown in Fig. 3b in the paper, the air-gap between two InP claddings often collapses due to the strain that was applied to the QW layer during the wafer growth. It is difficult to control the residual strain after the removal of QW. However, the air-gap collapsing does not degrade the *Q* factor. This is also well-analyzed in the Supplementary Reference [2]. Here, we present the calculated results in Supplementary Figure 4 to show that collapsing would actually reduce the optical loss, although its influence is not so significant on *Q* factor due to very small thickness (~ 20 nm) of the air-gap.

The calculations for the band-structure and thermal dissipation are presented in Supplementary Figure 5 and Supplementary Figure 6, respectively, to compare the two cases with and without the air-gap. Consequently, 20 nm is so thin that either its existence or collapsing does not significantly affect the characteristics of the device.

As shown in Supplementary Figure 5, the gap-collapsing does not change the TE-like band structure appreciably due to the direction of TE field that is tangential to the gap surface. Therefore, our TE-like cavity is hardly disturbed by the event of the 20 nm gap-collapsing, as long as we preserve the vertical symmetry of the structure in the fabrication.

Moreover, our calculations using a 3D finite volume method (COMSOL) in Supplementary Figure 6 show that this 20 nm air-gap practically does not make any difference in the thermal dissipation compared to the structure without the air-gap. This is because most of the heat generated in the active medium is always dissipated to the vertical directions through the thick InP claddings even when the air-gap is filled with InGaAsP QW. Here, the heat flux is shown in Supplementary Figure 6 with red arrows. With a few-μW heat source located in a 0.7×0.25×0.02 μm^3 active medium, we have observed that the temperature increase in either structure (with gap or without gap) has no practical difference. In experiments, the absorbed power in our nano-island QW laser is less than few μW during its operation.

Supplementary Note 4: Estimation of the β-factor using the conventional rate equations

In order to estimate the spontaneous emission factor β of our device, the experimental data for the 0th order mode in Fig. 4a in the paper is fitted to the conventional rate equations. In the equations, carrier density *N* and photon density *P* that reach steady states are described as follows:

$$\frac{dN}{dt} = \eta \frac{L_{\mathrm{in}}}{\hbar\omega_{\mathrm{p}} V_{\mathrm{a}}} - \left(\frac{A_{\mathrm{a}}}{V_{\mathrm{a}}} \nu_{\mathrm{s}} N + BN^2 + CN^3\right) - \Gamma G(N)P = 0 \quad (4)$$

$$\frac{dP}{dt} = \Gamma G(N)P - \frac{P}{\tau_{\mathrm{p}}} + \beta BN^2 = 0 \quad (5)$$

The definitions of the parameters and their numerical values are listed in Supplementary Table 1. Here we use the logarithmic gain $G(N) = \frac{g_0 c}{n_{\mathrm{eff}}} \ln\left(\frac{N}{N_{\mathrm{tr}}}\right)$ for QW as a function of the carrier density *N*. The frequency of pump laser ω_{p} and photon lifetime τ_{p} are given by $\omega_{\mathrm{p}} = \frac{2\pi c}{\lambda_{\mathrm{p}}}$ and $\tau_{\mathrm{p}} = \frac{\lambda_{\mathrm{out}} Q}{2\pi c}$, respectively. The spontaneous emission factor β, and the absorption ratio η of the pump laser in the active region are included in the fitting parameters in the Equations (4) and (5). As a result, the output power $L_{\mathrm{out}} = \frac{\hbar\omega_{\mathrm{p}} P V_{\mathrm{a}}}{\tau_{\mathrm{p}}}$ is plotted as a function of the absorbed power ηL_{in}. Fig 4b in the paper shows the logarithmic plot of ηL_{in}- L_{out} curve calculated from the rate equations with different values of β. The best fitting is obtained when $\beta \approx 0.43$ and $\eta \approx 0.05$.

Supplementary References

[1] Matsuo, S.; Shinya, A.; Kakitsuka, T.; Nozaki, K.; Segawa, T.; Sato, T.; Kawaguchi, Y.; Notomi, M.; "High-speed ultracompact buried heterostructure photonic-crystal laser with 13 fJ of energy consumed per bit transmitted," *Nat. Photonics* **4**, 648-654 (2010), DOI:10.1038/nphoton.2010.177

[2] Midolo, L.; Yoon, S. N.; Pagliano, F.; Xia, T.; Otten, F. W. M.; Lermer, M.; Höfling, S.; Fiore, A.; "Electromechanical tuning of vertically-coupled photonic crystal nanobeams," *Opt. Express* **20**, 19255-19263 (2012), DOI: 10.1364/OE.20.019255
